# Microwave ablation vs. surgery for T1bN0M0 papillary thyroid carcinoma: a propensity score-matched cohort study

**DOI:** 10.3389/fendo.2025.1671046

**Published:** 2026-01-14

**Authors:** Xinshu Zhao, Peiwen Wang, Deng-Ke Teng

**Affiliations:** Department of Ultrasound, China-Japan Union Hospital of Jilin University, Changchun, Jilin, China

**Keywords:** microwave ablation, papillary thyroid carcinoma, propensity score-matched cohort, surgery, treatment

## Abstract

**Objective:**

To assess the efficacy and safety of microwave ablation (MWA) in treating T1bN0M0 (T1b) papillary thyroid carcinoma (PTC) by comparing patient outcomes with those following surgical resection in treating T1b PTC.

**Methods:**

In this retrospective analytical study, patients who were diagnosed with T1b PTC in the ultrasound department of our hospital between April 2019 and October 2023. The patients were divided into MWA group and surgery group according to the different treatment methods. A 1:1 propensity score matching (PSM) analysis was performed to compare local tumor progression (LTP), relapse-free survival (RFS), and complication rates between patients treated with MWA and surgical resection.

**Results:**

After 1:1 PSM accounting for sex, age, nodule location, position and follow-up time as potential confounders, 172 patients with T1b disease were matched with 1:1 PSM, 86 patients each in the surgery and MWA treatment groups. Following PSM, there was no statistically significant difference in LTP (2.33% vs. 3.49%, p = 0.650) between the MWA group and the surgical group. The volume reduction rate (VRR) was 98.53 ± 6.50%. No permanent complications were observed after MWA, and no cases of distant metastasis or delayed surgery occurred.

**Conclusions:**

MWA could serve as a reasonable alternative besides surgery for T1b patients.

## Introduction

In recent years, the widespread application of high-resolution ultrasound and puncture biopsy has resulted in an increase in the detection rate of thyroid cancer (1). According to 2020 epidemiological statistics, the incidence of thyroid cancer is the ninth highest worldwide (2–4). Papillary thyroid carcinoma (PTC) is the most common subtype of thyroid carcinoma (5), and most patients are in the T1 stage of the disease. According to the guidelines of the American Thyroid Association (ATA) (6), the recommended method for treating T1 PTC is surgical resection. However, removal of the majority of the thyroid gland can cause changes in thyroid function and lead to the lifelong use of thyroid drugs (7), which can seriously affect the quality of life of the patients. For the above reasons, thermal ablation has become an important alternative for the treatment of T1-stage PTC because of its minimal invasiveness and precise therapeutic effects. The 2025 ATA Guidelines explicitly include percutaneous ablation as a therapeutic option (6). Previous studies have established the long-term efficacy of thermal ablation in the treatment of stage T1a PTC, and the European Thyroid Association recommends this technique as a suitable option for managing the disease (8). Although a small number of studies have evaluated the safety and efficacy of T1bN0M0 PTC (9–12), few studies have compared these characteristics simultaneously with those of surgical resection in the treatment of T1b PTC.

Therefore, the purpose of this study was to evaluate and compare the efficacy and safety of MWA with those of surgical resection in the treatment of T1b PTC.

## Materials and methods

### Patients

The data of patients with PTC who underwent MWA at the Ultrasound Department of China-Japan Union Hospital of Jilin University between April 2019 and October 2023 were retrospectively reviewed. Patients with a maximum PTC diameter between 10 mm and 20 mm were included in this study. All patients and their families provided informed consent for this study, which was reviewed and approved by the ethics committee of our hospital (2018110604).

Inclusion criteria:

1. Preoperative diagnosis of PTC confirmed by cytology or histopathology; the 2017 Bethesda System was used for FNAC classification (13).(2) No evidence of extrathyroidal extension (capsular interruption or involvement of surrounding tissues), local lymph node metastasis, or distant metastasis; the staging corresponds to cT1bN0M0.
2. Maximum lesion diameter between 10 mm and 20 mm;
3. Normal preoperative thyroid function and no history of other thyroid diseases (such as acute or chronic thyroiditis);
4. Follow-up duration of more than 12 months.

Exclusion criteria:

1. Presence of extrathyroidal extension, cervical lymph node metastasis, or distant metastasis;
2. Severe coagulation dysfunction, severe comorbid systemic diseases or immune-related disorders;
3. Insufficient follow-up information;
4. A history of previous thyroid surgery.

### Pretreatment evaluation

Prior to treatment, all patients underwent a comprehensive examination that included routine ultrasound to assess tumor size, morphology, and location, among other characteristics. Ultrasound-guided fine-needle aspiration (FNA) biopsy was performed to confirm the pathological classification of the tumor as papillary carcinoma. A 2 ml dose of SonoVue (Bracco Company, Italy) was injected into the patient via the median cubital vein, and contrast-enhanced ultrasound was subsequently performed to assess the margin and blood supply of the tumor. The tumor volume was calculated using the following equation: V = πabc/6 (V, volume; a, largest diameter on ultrasound; b and c, the diameters perpendicular to a). Other routine preoperative tests included blood tests, coagulation function tests, electrocardiography, and thyroid function tests. Additionally, detailed preoperative communication was conducted to ensure that the patients and their families understood the purpose of the surgery and the potential risks, and a surgical informed consent form was signed.

### Ablation procedure

All MWA procedures were performed by two experienced ultrasound physicians, one with over 5 years of ablation experience and the other with more than 10 years of ablation experience. The patients were placed in a supine position with their necks fully exposed. Under ultrasound guidance, local infiltration anaesthesia involving 1% lidocaine hydrochloride was administered percutaneously. The MWA system consisted of a 16G internally cooled MWA shaft antenna (3 mm) and an MWA therapeutic device (ECO-100C, Nanjing Yigao Company, China), which is consistent with our previous study (14). Real-time ultrasound scanning was performed with a Resona 7 color ultrasound diagnostic system (Mindray, China) equipped with a 5–14 MHz linear array probe. A 16G sharp needle measuring 4 cm in length was used to puncture the skin. The microwave electrode was subsequently inserted into the target area through the tunnel formed after removal of the sharp needle (15). The ablation power was set to 25 W. A combination of the fixed needle technique and the moving needle technique was used to perform the ablation, allowing the lesion and surrounding tissues to be ablated point by point and layer by layer (14). To reduce the risk of marginal residue and recurrence, an expanded ablation approach was adopted to ensure that the ablation zone extended at least 2 mm beyond the tumor margin whenever possible. For lesions adjacent to critical structures or tissues such as the carotid artery, trachea, oesophagus, or recurrent laryngeal nerve, a 20 ml syringe was used to inject saline into the surrounding tissue space for hydrodissection, forming a “liquid isolation zone” of at least approximately 5 mm to prevent thermal injury to these structures. Immediately after the procedure, contrast-enhanced ultrasound was performed to evaluate the ablation area and determine the presence of active bleeding.

### Surgery

Under general anaesthesia, thyroid surgery was performed by a surgeon with over 10 years of experience in thyroid surgery. The decision to perform total thyroidectomy, unilateral lobectomy with isthmusectomy, or partial thyroid lobectomy was made in accordance with the 2015 ATA guidelines (16) and the patient’s preferences(The patients enrollment occurred before the release of the 2025 guidelines).

### Postablation evaluation and follow-up

In the MWA group, follow-ups were conducted at 1, 3, 6, and 12 months postoperatively and every 6–12 months thereafter. Ultrasound imaging was used to assess the size, volume, and blood flow of the ablation zone and to identify any tumor recurrence and lymph node metastasis (LNM). Additionally, thyroid function tests were performed before and one month after the MWA procedure. The formula for calculating the volume reduction rate (VRR) was as follows: VRR = (Initial Volume - Final Volume) × 100%/Initial Volume). If tumor recurrence or LNM was suspected, needle biopsy was performed in the suspicious area. Recurrent tumors or LNMs were treated with additional ablation therapy or surgery according to the patient’s preference. Chest CT imaging was conducted annually to rule out distant metastasis. If symptoms of distant metastasis were noted, positron emission tomography (PET) or bone scanning was performed.

In the surgery group, follow-ups were conducted every 6 to 12 months postoperatively and consisted of ultrasound, thyroid function tests, and chest CT scans (annually).

### Endpoints and definitions

The primary outcomes were local tumor progression (LTP), distant metastasis, and recurrence-free survival (RFS). LTP was defined as (17): (1) biopsy-confirmed LNM confirmed; (2) secondary lesions at sites distant from the ablated tumor, confirmed as PTC by biopsy; and (3) persistent presence of the tumor at the ablation site as confirmed by biopsy. Distant metastasis was defined as suspicious metastatic lesions detected by CT, PET, or bone scanning. RFS was defined as the time from the start of treatment to tumor recurrence or the date of the last follow-up.

The secondary outcomes were treatment VRR and complications. According to the standards for image-guided thyroid ablation and surgery (18, 19), complications were categorized into major complications (i.e., recurrent laryngeal nerve injury, permanent hypoparathyroidism, and airway obstruction), minor complications (i.e., minor haematoma), and side effects (i.e., transient hypoparathyroidism). Recurrent laryngeal nerve paralysis or hypoparathyroidism lasting >6 months was defined as a permanent complication.

### Statistical analysis

Data processing was performed using SPSS version 25.0. Continuous data are expressed as the means ± standard deviations, and categorical data are presented as frequencies and percentages. To account for potential biases inherent in this retrospective analysis, 1:1 propensity score matching (PSM) was applied, accounting for sex, age, nodule location, and follow-up duration as potential covariates. The chi-square test or Fisher’s exact test was used for intergroup comparisons. Independent samples t tests were used to compare continuous data with a normal distribution and homogeneity of variance, whereas the Mann–Whitney U test was used for continuous data with a nonnormal distribution or heterogeneity of variance. Kaplan–Meier analysis was conducted to generate RFS curves, and comparisons were made using the log-rank test.

## Results

After 1:1 PSM with sex, age, nodule location, position and follow-up duration as potential confounders, a total of 172 patients with T1b stage disease were similarly matched with 1:1 PSM, with 86 patients each in the surgery and MWA groups. It should be noted that during the matching process, we defined nodules position with distances exceeding 2mm from the capsule as central positions, while those within 2mm were classified as peripheral positions. The average follow-up durations for the MWA and surgery groups were 21.77 ± 11.27 months (range: 12–62 months) and 22.40 ± 11.21 months (range: 12–64 months), respectively. There were no significant differences in baseline characteristics between the two groups; other relevant characteristics are presented in **Table 1**.

**Table 1:** Baseline characteristics of surgical and MWA treatment for patients in stage T1b.

Variable	T1b after propensity score matching		
	MWA	Surgery	*P*
	n=86	n=86	
Age (years)	41.06±12.47	41.44±10.63	0.828
Sex			0.858
Female	66	65	
Male	20	21	
Follow-up period (months)	21.77±11.27	22.40±11.21	0.715
maximum diameter (cm)	1.28±0.26	1.30±0.25	0.597
Location			0.925
Left lobe	40	39	
Right lobe	43	43	
Isthmus	3	4	
Position is in the center or on the edge			0.644
Central position	49	46	
Peripheral position	37	40	
Laboratory studies, mean ± SD			
TSH (mIU/l)	2.12±1.49	2.45±1.36	0.127

### Primary outcomes

The local tumor progression (LTP) rate was 3.49% (3/86) in T1b patients following MWA. The oncologic outcomes included a local tumor recurrence rate of 2.33% (2/86), a lymph node metastasis (LNM) rate of 1.16% (1/86), and no cases (0%) of lesion persistence. All three patients with LTP (including those with recurrence or LNM) were successfully managed with repeat MWA, achieving complete lesion inactivation. No distant metastases occurred during follow-up, and no surgical intervention was required for LTP or patient anxiety regarding the procedure.

Among T1b patients treated with surgery, the overall incidence of LTP was 2.33% (2/86). Among these patients, the overall incidences of LNM and recurrent PTC were 1.16% (1/86) and 1.16% (1/86), respectively. Among the patients who underwent surgery, one case presented with lymph node metastasis in the left (side of the lesion) neck level III. Another case experienced recurrence, in which a new malignant lesion was identified in the contralateral lobe following an ipsilateral lobectomy. After PSM, there were no significant differences between surgical resection and MWA treatment for T1b patients in terms of the incidence of LTP (2.33% vs. 3.49%, p = 0.650), LNM (1.16% vs. 1.16%, p = 1.000), or recurrent PTC (1.16% vs. 2.33%, p = 0.560).

**Figure 1:**
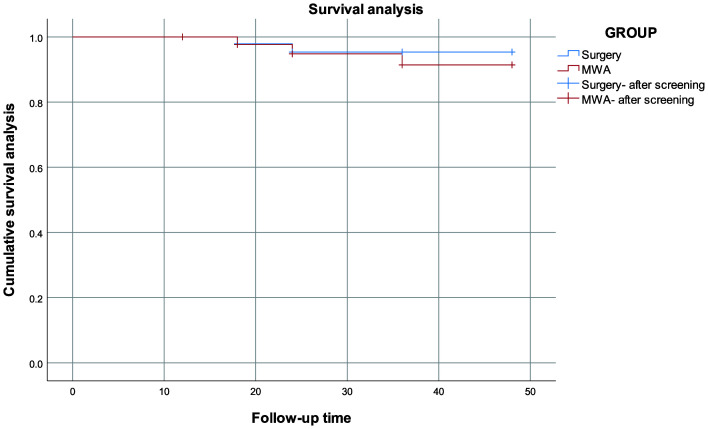
Comparison of survival analysis between MWA group and surgical group.

After PSM, the RFS rates for T1b PTC patients treated with surgical resection and MWA were 97.67% and 96.51%, respectively. No significant difference was observed in the RFS rates between the two groups (P > 0.05) (**Figure 1**).

### Secondary outcomes

#### VRR

**Figure 2:**
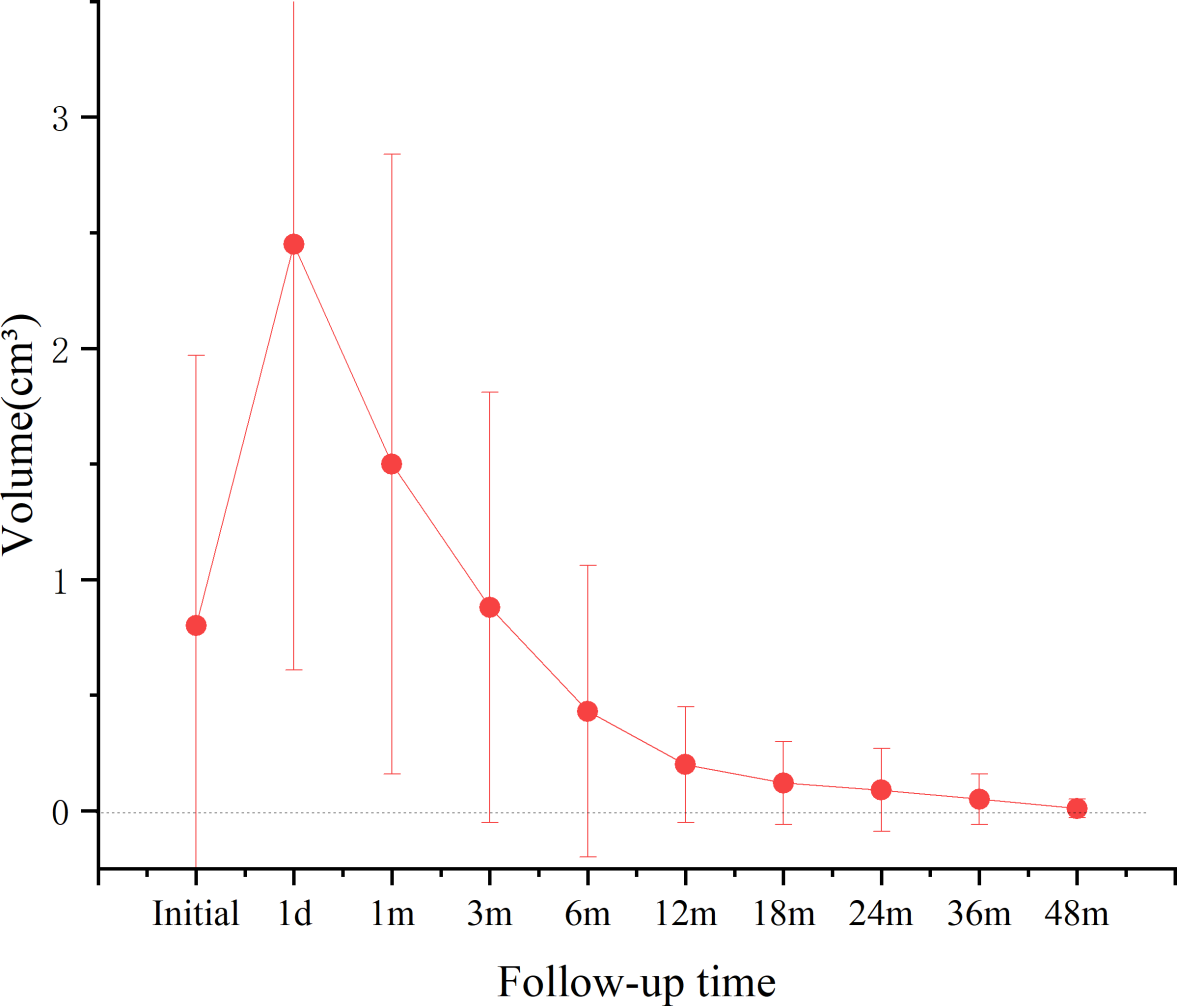
Volume change of MWA group ablation area.

**Figure 3:**
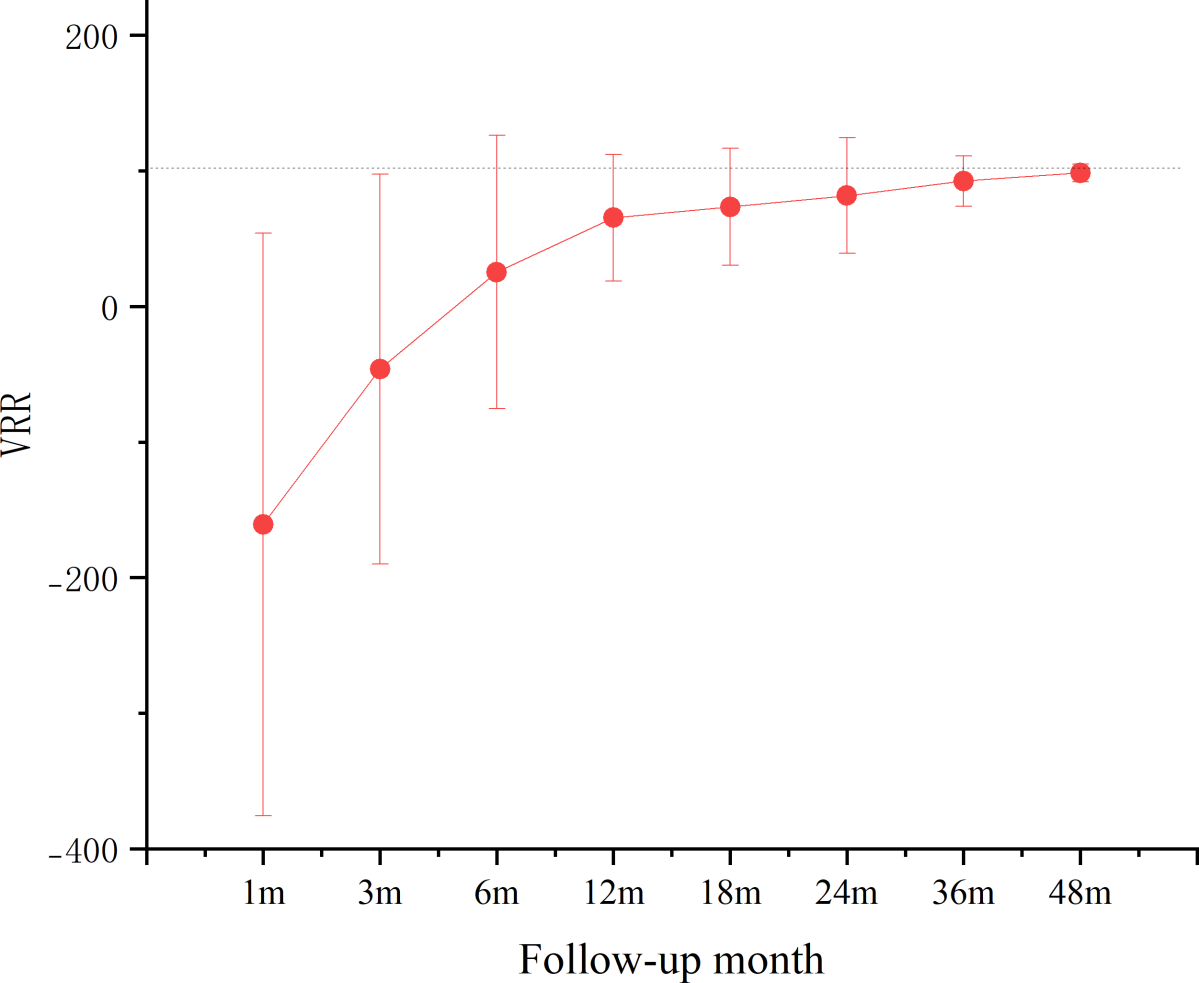
VRR changes of MWA group.

During the follow-up period, the volume of the ablation area significantly decreased. At the last follow-up, the VRRs for T1b groups was 98.53%. The rate of complete tumor disappearance was (42/83, 50.60%). The changes in volume of the ablation area during follow-up are shown in **Figure 2**, and the changes in VRR are shown in **Figure 3**.

### Complications

MWA and surgery was considered successfully completed for all patients. After PSM, the overall complication rate was 15.1% (26/172), and the major complication rate was 1.81% (3/166). In the T1b group, 12.79% (11/86) reported pain, and 2.33% (2/86) patients experienced recurrent laryngeal nerve injury, both of which resolved within 3 months. In the surgery group, 8.14% (7/86) developed postoperative fever, with the longest lasting up to 4 days, and 6.98% (6/86) experienced recurrent laryngeal nerve injury, 5 of which resolved within 1–3 months, but one patient (1.16%, 1/86) developed persistent recurrent laryngeal nerve injury, which is considered to be permanent recurrent laryngeal nerve injury, and furthermore, laryngoscopy confirmed limited vocal cord mobility on the affected side. For the patients with recurrent laryngeal nerve injury, a laryngoscopy was performed the day after surgery or MWA treatment. The laryngoscopy reveals varying degrees of vocal cord mobility restriction in these patients. There was no significant difference in the incidence of major complications between surgery and MWA among T1b patients (1.16% vs. 2.33%, p = 0.688).

## Discussion

Currently, T1 stage PTC, a malignant tumor with a high incidence but low mortality, is often treated surgically. Given the significant trauma and high rate of postoperative complications associated with surgery, many scholars have recently turned their attention to investigating outcomes of low-risk PTC patients under Active Surveillance (AS) (20, 21). However, evidence from some studies indicates that during AS, a portion of patients opt for surgical treatment due to disease progression or anxiety. A Japanese study revealed that T1bN0M0 PTC patients managed with AS experienced increases in tumor size and volume, with a 10-year tumor progression rate of 12% (22). Another study revealed that approximately 13% of patients managed with AS underwent thyroid surgery, with 29% of these patients developing LNM (23). Therefore, most scholars and patients advocate for better treatment approaches that can effectively manage the disease while avoiding excessive harm, and thus achieving a reasonable balance between surgery and AS has become an important direction in the treatment of PTC. MWA is a minimally invasive treatment that has been widely applied for PTC over the past decade, particularly for T1aN0M0 stage tumors (24). Although the 2025 ATA guidelines include cT1aN0M0 stage in the MVA treatment regimen, cT1bN0M0 stage PTC is not recommended. This is because there is still insufficient evidence regarding the efficacy of MWA for treating cT1bN0M0 PTC (4). Key questions, such as whether MWA for T1bN0M0 patients can achieve comparable outcomes to surgical treatment remain unanswered. Therefore, this study sought to obtain evidence to address these questions by comparing the clinical outcomes of T1b PTC patients treated with MWA with those of surgical resection via a PSM analysis. The results suggest that MWA may be a feasible and safe alternative for treating patients with T1bN0M0 PTC.

By comparing tumor progression in patients with T1bN0M0 PTC, the results of this study revealed that the efficacy of MWA in treating T1b PTC was similar to that of surgical resection. Specifically, there were no significant differences in LTP (2.33% vs. 3.49%, p = 0.650) or RFS (97.67% vs. 96.51%, p = 0.581) between T1b TPC patients treated with surgical resection and MWA treatment, consistent with the results of previous comparative studies (3, 25). The beneficial effects of MWA in the treatment of T1b may be due to the accumulation of sufficient experience and technological advancements in the treatment of T1a PTC as well as the accumulation of experience in the treatment of T1b PTC. Although stage T1b PTC patients present with large tumors, extended ablation ensures a sufficient safety margin. At present, immediate postoperative contrast-enhanced ultrasound can be used to observe the internal and surrounding conditions of the tumors to ensure that the PTC is completely inactivated.

This study revealed that there was no significant difference between the groups in terms of complications. First, although there was no statistically significant difference in the incidence of major complications between MWA and surgical treatment for T1b PTC (2.33% vs. 6.98%, p = 0.148), the incidence of complications was lower in the MWA group. Compared with traditional surgery, MWA is less invasive and avoids the extensive tissue damage caused by open surgery; thus, patients treated with MWA had a lower complication rate. Second, in most patients managed with MWA, recurrent laryngeal nerve injury is due to temporary nerve dysfunction caused by heat conduction rather than permanent damage to nerve structures. This type of heat injury usually manifests as nerve oedema and reversible conduction block, and patients can achieve varying degrees of neurological functional restoration after 3–6 months (26).

Although MWA can successfully treat T1bN0M0 PTC and its clinical efficacy is similar to that of surgery, MWA may not be able to treat lesions that do not appear on US. As the follow-up time increases, unobserved lesions are more likely to develop into new PTCs or metastasize to the lymph nodes (27). These risks can be mitigated with total thyroidectomy or unilateral lobectomy. However, patients in this study who relapsed after MWA were treated with MWA a second time, which yielded good results during the follow-up period. These findings highlight the importance of careful examination using high-resolution ultrasound before MWA to detect small lesions and treat them all during ablation. In our study, although the difference was not significant, the incidence of LTP was slightly greater in the MWA group than in the surgery group. Therefore, during MWA, the extent of the ablation should be expanded to the normal thyroid parenchyma 2 mm around the tumor to reduce the risk of marginal residual and recurrent tumors.

There are several limitations to this study. First, the sample size is relatively small, and the follow-up period is not long enough to discuss long-term outcomes or altered recurrence-free survival rates. So, further large-scale multicenter studies over a longer follow-up period are needed. Second, this was a retrospective study, and future prospective and randomized controlled studies are needed to provide more convincing evidence of the efficacy and safety of the investigated treatment methods.

## Conclusion

This study revealed that MWA is a safe treatment for T1bN0M0 PTC patients and shows similar efficacy to MWA for treating T1aN0M0 PTC patients and surgical resection for treating T1bN0M0 PTC patients. MWA could therefore serve as a reasonable alternative for T1b patients.

## Data Availability

The raw data supporting the conclusions of this article will be made available by the authors, without undue reservation.
