# Correction: Microwave ablation vs. surgery for T1bN0M0 papillary thyroid carcinoma: a propensity score-matched cohort study

**DOI:** 10.3389/fendo.2026.1810475

**Published:** 2026-03-02

**Authors:** Xinshu Zhao, Peiwen Wang, Deng-Ke Teng

**Affiliations:** 1 Department of Ultrasound, China-Japan Union Hospital of Jilin University, Changchun, Jilin, China; 2 Future Medical Laboratory, The 2nd Affiliated Hospital of Harbin Medical University, Harbin, China

**Keywords:** microwave ablation, papillary thyroid carcinoma, propensity score-matched cohort, surgery, treatment

Affiliation “Department of Ultrasound, China-Japan Union Hospital of Jilin University, Changchun, Jilin, China” was omitted for author Xinshu Zhao. This affiliation has now been added for author Xinshu Zhao.

The original version of this article has been updated.

