# Correction: Microwave ablation vs. surgery for T1bN0M0 papillary thyroid carcinoma: a propensity score-matched cohort study

**DOI:** 10.3389/fendo.2026.1915207

**Published:** 2026-07-15

**Authors:** Xinshu Zhao, Peiwen Wang, Deng-Ke Teng

**Affiliations:** Department of Ultrasound, China-Japan Union Hospital of Jilin University, Changchun, Jilin, China

**Keywords:** microwave ablation, papillary thyroid carcinoma, propensity score-matched cohort, surgery, treatment

Author “Xinshu Zhao” was erroneously assigned to affiliation “Future Medical Laboratory, the 2nd Affiliated Hospital of Harbin Medical University, Harbin, China”. This affiliation has now been removed for author “Xinshu Zhao”. “Department of Ultrasound, China-Japan Union Hospital of Jilin University, Changchun, Jilin, China”. served as the first and sole affiliation for the author “Xinshu Zhao”.

The original version of this article has been updated.

